# Rapid mixed-methods assessment of COVID-19 impact on Latinx sexual minority men and Latinx transgender women

**DOI:** 10.1371/journal.pone.0244421

**Published:** 2020-12-31

**Authors:** Sarah MacCarthy, Max Izenberg, Joanna L. Barreras, Ron A. Brooks, Ana Gonzalez, Sebastian Linnemayr

**Affiliations:** 1 Behavioral and Policy Sciences, RAND Corporation, Santa Monica, California, United States of America; 2 Pardee RAND Graduate School, RAND Corporation, Santa Monica, California, United States of America; 3 School of Social Work, California State University, Long Beach, California, United States of America; 4 Bienestar Human Services, Inc., Los Angeles, California, United States of America; 5 Economics, Sociology, and Statistics, RAND Corporation, Santa Monica, California, United States of America; University of Toronto, CANADA

## Abstract

We conducted a rapid, mixed-methods assessment to understand how COVID-19 affected Latinx sexual minority men (LSMM) and transgender women (LTGW). Using a computer-assisted telephone interviewing software, one interviewer called 52 participants (randomly sampled from a larger HIV prevention pilot study aiming to increase HIV knowledge and testing frequency; n = 36 LSMM and n = 16 LTGW) between 04/27/20-05/18/20. We quantified core domains using the Epidemic-Pandemic Impacts Inventory scale and provided important context through open-ended qualitative questions assessing: 1) COVID-19 infection history and experiences with quarantine; 2) Health and healthcare access; 3) Employment and economic impact of COVID-19. Participants reported increases in physical conflict or verbal arguments with a partner (13.5%) or other adult(s) (19.2%) due to stressors associated with the safer-at-home order. Participants also reported increased alcohol consumption (23.1%), problems with sleep (67.3%) and mental health (78.4%). Further, disruptions in access to Pre-Exposure Prophylaxis or PrEP–a daily pill to prevent HIV–occurred (33.3% of 18 participants who reported being on PrEP). Many said they received less medical attention than usual (34.6%), and LTGW reported delays in critical gender-affirming hormones/procedures. Half of the participants lost their jobs (50.0%); many undocumented participants relayed additional financial concerns because they did not qualify for financial assistance. Though no COVID-19 infections were noted, COVID-19 dramatically impacted other aspects of health and overall wellbeing of LSMM and LTGW. Public health responses should address the stressors faced by LSMM and LTGW during the COVID-19 pandemic and the impact on wellbeing.

## Introduction

Lesbian, gay, bisexual, and transgender (LGBT) communities experience disproportionately high rates of HIV [1], cancer [2], and behaviors such as smoking that can lead to worsened health [3] that may increase their risk for serious complications once contracting COVID-19 [4, 5]. Additionally, with reduced access to critical health services during the COVID-19 pandemic, such as testing for HIV and sexually transmitted infections (STIs) [6], any STIs may remain undiagnosed and untreated, leading to critical delays in accessing treatment that could contribute to worse health outcomes [7, 8]. Further, disruptions in access to critical medications–such as Pre-Exposure Prophylaxis (PrEP–a daily pill that if taken correctly and consistently can reduce the risk of HIV from sex by 99%) [9], could further increase the risk of transmission among groups who are disproportionately impacted by HIV and STIs compared to the general population.

Data are still limited regarding the impact of COVID-19 on racial/ethnic minority groups [10]. The reasons underlying existing health disparities [11], as well as the potentially disproportionate burden of COVID-19 infections among racial/ethnic minorities, are complex and relate to individual (e.g., higher rates of underlying comorbidities) [12] and structural (e.g., crowded living conditions and lower-paying jobs that limit physical distancing) [13] factors that challenge the risk and response to COVID-19 [14]. Further, among immigrant Latinx populations, the Supreme Court ruling regarding the Inadmissibility on Public Charge Grounds [15] (which significantly limits who can qualify for permanent residency) has further exacerbated existing fears that engaging with healthcare could negatively affect their immigration status. In an effort to expand the data available, a broad coalition of elected officials introduced a bill requiring the U.S. Department of Health and Human Services to collect and report racial and ethnic data related to COVID-19 [16]. Still, substantial data lags challenge ongoing attempts to concretely identify the pandemic’s impact on vulnerable communities. It is likely that the barriers complicating the risks of and response to COVID-19 are even greater among racial/ethnic sexual and gender minority individuals, such as Latinx sexual minority men (LSMM) and Latinx transgender women (LTGW) [17]. For example, since sexual and gender minority populations lack federal employment protections they may be at elevated risk for unemployment, and especially if undocumented, may have no access to financial relief offered by the federal government [14]. Further, being uninsured or underinsured will continue to serve as a barrier to general health services, in addition to new concerns introduced by COVID-19 testing and treatment [18]. These intersecting forms of marginalization underscore the need to identify and address the ways in which COVID-19 impacts particularly vulnerable communities such as LSMM and LTGW.

On March 19^th^ 2020, Los Angeles Mayor Garcetti issued a “Safer at Home” (SAH) emergency order requiring “all residents to stay inside their residences and limit all movement outside of their home beyond what is absolutely necessary to take care of essential needs” [19]. To expand the existing base of knowledge, we conducted a rapid, mixed-methods assessment by phone to understand how COVID-19 was impacting the health and wellbeing of LSMM and LTGW living in Los Angeles County (LAC).

## Materials and methods

We used a mixed-method study design to quantify core domains and understand the frequency of responses, while also providing important context through open-ended qualitative questions [20, 21]. The following three core domains were examined quantitatively and qualitatively: 1) COVID-19 infection history and experiences with quarantine; 2) Health and healthcare access; 3) Employment and economic impact of COVID-19. We considered focusing only on LSMM to avoid combining populations with distinct needs based on sexual orientation and gender identity. However, information is urgently needed on both LSMM and LTGW, and the mixed-methods approach allows us to highlight the similarities and differences of these two vulnerable communities.

### Design

Phone interviewers were conducted by one interviewer (cisgender female, Latina, age 28, and three years of post-bachelor experience conducting qualitative interviews with Latinx populations). She had no previous relationship with the participants, though participants were familiar with the principal investigators of the study through the recently completed study funded by the National Institutes of Health [R34 MH109373] [22]. This parent study was a quasi-experimental pilot study (n = 218) using mobile health technology in combination with behavioral economics to increase HIV knowledge and testing frequency. The study included two intervention arms–the ‘Information Only’ group that received text messages with HIV prevention information and the ‘Information Plus’ group that additionally could win incentives by answering weekly quiz questions correctly and testing for HIV within a given three-month period. Results showed no effect on HIV knowledge in the ‘Information Only’ group but a statistically significant increase in the ‘Information Plus’ group. Further the frequency of HIV testing was higher for both intervention groups compared to a synthetic control group [23].

The results presented here are based on a study among a subset of participants from the parent study. We randomized the list of original participants (n = 166), then began contacting them in a random order until the SAH orders were amended. We decided to halt data collection once the SAH orders were lifted to allow for increased access to services and other social gatherings, and likely impacting the response of participants regarding the impact of COVID-19 on their health and wellbeing. Of those contacted (n = 133), nearly half did not respond to either being called or texted (n = 64), few responded but declined (n = 9) or had phone numbers that were no longer in service (n = 7). At the time that the SAH was lifted, we had achieved 52 interviews. All participants were originally recruited through our partner Bienestar, Human Services Inc. (hereafter Bienestar) a community-based organization with six offices across LAC. Bienestar is focused on serving Latinx communities including HIV-positive people, individuals at risk for HIV, LGBT, youth and people who use substances.

### Participants

We interviewed a total of 52 participants (n = 36 LSMM and n = 16 LTGW), drawing from the parent study’s sample. Criteria for inclusion were the same as those from the parent study including: being HIV negative; self-identifying as SMM or TGW as well as Latinx; 18 years of age or older; owning or having regular mobile phone access; and fluency in English or Spanish. Comparing mean difference in age and proportions of Spanish-language preference, education level, income, employment status, or citizenship status, we found no statistical differences between the current study sample and the sample of the larger parent study.

### Procedures

Institutional Review Board approval was received for the study protocols and materials (RAND Human Subjects Protection Committee and The Los Angeles County Public Health, Ambulatory Care network and Health Services Administration Institutional Review Board). Data was collected between April 27 –May 18, 2020. We randomized the participant list from the completed study to contact participants. Interviews were conducted by phone in the participant’s preferred language (either Spanish or English) by a Latinx bilingual study coordinator. All participants were read a copy of the informed consent and provided verbal consent. The interviewer noted that verbal consent was attained before initiating the survey. The interview lasted between 20 and 30 minutes. The interview began with socio-demographic questions (Table 1), followed by quantitative questions regarding the impact of COVID-19 on health and wellbeing. The quantitative questions were based on the Epidemic–Pandemic Impacts Inventory [EPII], a tool designed to assess tangible impacts of COVID-19 across personal and social life domains. All candidate items from the EPII were constructed and tested by a team of clinical and developmental psychologist with feedback from professionals across multiple disciplines and finalized via expert consensus (Table 2) [24]. Following the quantitative questions, the interview included questions regarding PrEP disruptions [25] associated with COVID-19 (Table 3). The interview asked qualitative questions to provide context and nuance for the quantitative responses and highlight similarities and differences between LSMM and LTGW (Table 4). The complete interview scripts were programmed using Select Survey (a web-based survey software which can be used for computer-assisted telephone interviewing—CATI). Participants received a $15 electronic gift card via email or text message for participating in the interview. Additionally, participants were emailed a COVID-19-related resource sheet with LGBT-specific information.

**Table 1:** Socio-demographic characteristics of Latinx sexual minority men and transgender women (n = 52).

	Latinx Sexual Minority Men (n = 36)	Latinx Transgender Women (n = 16)	Total (n = 52)
*Background Characteristics*			
Mean age	40.2	39.4	39.9
At least a high school education	80.6	50.0	71.2
Full-time employed	63.9	43.8	76.9
Annual income less than $35,000	22.2	6.3	17.3
*Relationship Status*			
Single	66.7	73.3	68.6
Married	11.1	6.7	9.8
Living with a partner	19.4	20.0	19.6
Divorced, separated, or widowed	2.8	0.0	2.0
*Documentation Status*			
U.S. citizen or permanent resident	63.9	50.0	59.6
Deferred Action for Childhood Arrivals (DACA), asylum, and U visas for victims of violent crimes	5.6	25.0	11.5
Undocumented	22.2	18.8	21.2
Other	8.3	6.3	7.7

**Table 2:** Responses to the Epidemic–Pandemic Impacts Inventory (EPII) among Latinx sexual minority men and transgender women (n = 52).

	Percentage of Respondents			
	Yes (Me)	Yes (Person in home)*	No	N/A**
*COVID-19 Infection History and Experiences with Quarantine*				
Had symptoms of this disease but never tested.	15.7	0.0	80.4	3.9
Currently have symptoms of this disease but have not best tested	3.9	0.0	92.9	3.8
Tested and currently have this disease.	0.0	0.0	92.2	7.8
Tested positive for this disease but no longer have it.	0.0	0.0	96.2	3.8
Isolated or quarantined due to symptoms of this disease	5.8	0.0	94.2	0.0
Isolated due to existing health conditions that increase risk of infection or complications from this disease	7.7	1.9	92.3	9.0
Increase in physical conflict or verbal arguments with a partner	13.5	0.0	84.6	1.9
Increase in physical conflict or verbal arguments with other adult(s) in home	19.2	0.0	80.8	0.0
*Health and Healthcare Access*				
Increase in use of alcohol	23.1	1.9	75.0	0.0
Increase in use of substances	3.9	0.0	96.1	0.0
Increase in mental health problems or symptoms (e.g., mood, anxiety, stress, depression)	78.4	17.7	21.6	0.0
Increase in sleep problems or poor sleep quality	67.3	11.5	32.7	0.0
Unable to get needed medications (e.g., prescriptions)	13.5	0.0	86.5	0.0
Got less medical care than usual (e.g., routine or preventive care appointments)	34.6	1.2	65.4	0.0
*Employment and Economic Impact of COVID-19*				
Laid off from job or had to close own business	50.0	15.4	36.5	3.9
Reduced work hours or furloughed	57.7	15.5	25.0	11.5
Had to continue to work even though in close contact with people who might be infected (e.g., customers, patients, co-workers)	34.6	5.8	50.0	9.6
Increase in workload or work responsibilities	30.8	3.9	57.7	7.7
Unable to get enough food or healthy food	36.5	1.9	63.5	0.0
Unable to pay important bills like rent or utilities	55.8	5.8	44.2	0.0

*Person in the home–this response category was deemed important given the risk of infection due to shared living space as well as due to the shared impact of social and/or economic consequences of COVID-19.

**Per guidance read to the interviewee, N/A was used if the statement did not apply to the interviewee or someone in the home.

***As respondents can state that both they and someone in their home may be impacted, in some instances, row summaries can be greater than 100%.

**Table 3:** PrEP disruptions due to COVID-19 among Latinx sexual minority men and transgender women (n = 52).

	N	%
Previously on PrEP*	18	35.6
Have not yet reached out for a refill	9	50.0
Reached out for a refill (n = 9)		
With telemedicine appointment	5	55.6
With office-based medical visit	4	44.4
Continued on PrEP**	12	66.7
Concerned about access to PrEP since Stay at Home Order? (n = 12)		
Not concerned or not concerned very much	8	66.7
Neutral	1	8.3
Concerned or very concerned	3	25.0

*18 out of 52 participants responded ‘yes I was previously on PrEP’.

**12 out of the 18 participants who reported previously being on PrEP responded ‘yes I have continued on PrEP’.

**Table 4:** Exemplary quotes highlighting similarities and differences between Latinx sexual minority men and transgender women (n = 52).

Main Theme	Sub Theme	Latinx Sexual Minority Men	Latinx Transgender Women
**COVID-19 Infection History and Experiences with Quarantine**	Willingness to seek care with COVID-19 symptoms	". . . I do believe that there is some programs and it does not matter if you don’t have medical insurance, they will still assist you." (age 29, asylum seeker)	"Of course. I would take care because it impacts all people. If I felt that I had it, I would seek treatment and I would go because this is a serious problem and not trivial." (age 53, permanent resident)
	Feeling safe as an LGBT person in quarantine	"Well I think by staying home, we are safer, and not only safe but healthy. Better than being out in the streets. . . I feel very safe and secure because all my family is accepting of me, and they have accepted me as I am." (age 42, permanent resident)	"Well I feel safe because, I can protect my health, if it is true about the number of infections, then I can prevent mine by staying home, and ensure my health." (age 40, asylum seeker)
**Health and Healthcare Access**	Impact on Health	“Well, yes like a depression being enclosed in quarantine because we would like to be able to go out a little on weekends, and before the weekend there is a significant amount of stress because of work, all with a little stress, and the weekends are supposed to be relaxing. This has changed. The work, being at home, and little by little and all this creates depression because there is also the lack of social contacting, and for this I think COVID-19 really has affected me.” (age 29, asylum seeker)	“What is going on with COVID there are times that this produces, anxiety, concern, it causes low moods, because just with the simply act of thinking of it, I experienced a back pain and then I associated it with COVID and I thought I had it, and the first thing that came to my mind hence all the things that I see on TV was what if I die? I do not want to die, and it is really is killing many people and so all this created so much anxiety and worry, that all I wanted to do was to run to get tested so that I can confirm I do not have it, and get out of this doubt I have, I could not sleep for two or three days.” (age 37, permanent resident)
	Access to PrEP	"Yes, I was but I was going to get my pills but I’m not going to… because of the [stay at home order] and because I’m not busy, and I don’t have anything going on, I have even become abstinent." (age 54, undocumented)	"What happen is that I finished my prescription, and I have not been able to get a refill. I don't recall the specifics, but it was something with my insurance, that they were not able to provide me with a refill" (age 36, undocumented)
	HIV/STI Testing	Not fearing the risk of COVID-19 to get tested: "I feel comfortable and secure getting tested frequently." (age 52, undocumented)	Not fearing the risk of COVID-19 to get tested: ". . . I had an appointment to get my tests done like HIV and STI's and I went and I felt comfortable, in the entrance they checked my fever, the guy that did so was extremely protected, the reception desk looked very organized, in the lobby waiting room social distancing was being practiced, and there were a few people, about 3 to 4 people there, until I entered to see my doctor, and I felt fine because everything seemed very protective. " (age 40, asylum-seeker)
		Fearing the risk of COVID-19 to get tested: "I’m not going to any clinic, primarily because I am sexually in quarantine [laughs] and additionally, because I’m afraid that maybe, I think that going to a place like that, you have a higher risk of infection, so that when weighing it all out, I don’t feel safe going to a clinic at this time." (age 57, permanent resident)	Fearing the risk of COVID-19 to get tested: "I feel good about getting tested, but then the doubts that I have of the reality of this virus I would have doubts and I don't know if I would still go. I think of the fear that all this is true and then become infected from COVID." (age 29, Permanent Resident)
	Unique concerns regarding gender-affirming care for LTGW		". . .I had approval to do the facial feminization and hair removal (face, mustache and beard, intimate parts), my appointments have been delayed and they don’t know when I will get another appointment, until things are more calm with coronavirus… sometimes, so I can’t see my doctor to see my levels of hormones, and they can’t see me because they are not doing check-ups… they can’t check my blood well because they are not making lab [appointments] because they aren’t receiving patients–it has affected my health in that way. . ." (age 50, work permit)
**Employment and Economic Impact of COVID-19**		"I think more in thinking about the future, like, for instance the economy. . .Everything is like, uncertain. It’s uncertain, you know? So that creates a bit of anxiety which has been, of course, I want to control that by having a drink, you know? I know it’s not the best medicine, and I’m aware of that. . ." (age 51, U.S. Citizen)	"… this pandemic came to change everyone’s life. We are all in a dire situation, with late bills, no job, and instead of feeling safe at home what happens you feel insecure at home because we all know very well that that when this ends, as a result people will be kicked out of their homes… and for all that help the government is giving out I have not received not one dollar again as I told you I do not have the correct documentation. So, then I cannot say “oh I was sent $1,200 dollars, I received 800 or 600 from unemployment, because I can’t. I have received nothing!" (age 54, DACA)

### Data analysis

Interviews were linked to information from the parent study to obtain additional sociodemographic information. Survey responses from the CATI were imported into Stata version 15.1. Data were coded, cleaned, and then analyzed using basic descriptive statistics. Due to the small sample size and general shared concern about COVID-19 risks across both groups, we did not test for differences between LSMM and LTGW; in most cases quantitative results were substantially similar and we therefore report the aggregate responses.

For the qualitative analysis, all qualitative interviews (n = 52) were recorded, transcribed and when necessary translated. The transcripts were de-identified and then uploaded into Dedoose (Version 8.3.21). A draft codebook was developed that was iteratively revised. To establish inter-rater reliability, two team members (SM and MI) coded a randomly selected set of 21 transcripts representing approximately 40% of total excerpts (n = 170). Pooled Cohen’s Kappa was 98%, indicating “good agreement” between reviewers, and one team member (SM) coded the remaining interviews [26]. We used a directed content analysis [27] whereby coding categories were based on the domains of interest including 1) COVID-19 infection history and experiences with quarantine; 2) Health and healthcare access; 3) Employment and economic impact of COVID-19. The qualitative results provided further context to the quantitative responses and highlighted similarities and differences between LSMM and LTGW participants.

## Results

The sociodemographic characteristics for LSMM and LTGW are reported in Table 1. More than two-thirds (69.0%) self-identified as being a sexual minority man (n = 36) and about a third (31.0%) self-identified as being a transgender woman (n = 16). LSMM and LTGW were similar across most sociodemographic characteristics such as age (mean age of 40.2 for LSMM vs. 39.4 for LTGW) and relationship status, as most reported being single (66.7% of LSMM vs. 73.3% of LTGW) or living with a partner (19.4% of LSMM vs. 20.0% LTGW). There were differences with respect to having at least a high school education (80.6% of LSMM vs. 50.0% of LTGW), being employed full-time (63.9% of LSMM vs. 43.8% of LTGW), and reporting an annual income greater than $35,000 (22.2% of LSMM vs. 6.3% of LTGW). While LSMM and LTGW participants reported similar percentages of U.S. citizenship or permanent residency (63.9% of LSMM vs. 50.0% of LTGW) and undocumented residency status (22.2% LSMM vs. 18.8% of LTGW), far more LTGW reported their documentation status as Deferred Action for Childhood Arrivals (DACA), asylum, or U visas for victims of violent crimes (25.0%) compared to LSMM (5.6%). Additionally, 21.2% of participants reported having at least one chronic health condition and 26.0% of participants consider themselves regular smokers–two characteristics known to increase the likelihood of complications if infected with COVID-19 [28].

Below, we report the quantitative results for each domain (Tables 2 and 3) and then complement it with qualitative findings (Table 4).

### COVID-19 infection history and experience with quarantine

Quantitative responses to the EPII survey module (Table 2) show that few participants reported either currently (3.9%) or previously (15.7%) having symptoms of the disease, and no participants reported having been tested for COVID-19. Few reported going into isolation or quarantine specifically due to having symptoms of the disease (5.8%) or due to existing health conditions that increased their risk of complications if infected (7.7%). Participants stated an increase in physical conflict or verbal arguments with a partner (13.5%) or with other adult(s) in their home (19.2%).

The qualitative results (Table 4) indicate almost all participants said they were following the SAH orders. All but one participant said they would seek care if they felt they had COVID-19. When probed about potential barriers to seeking care (e.g., documentation status or not having health insurance), participants often relayed that they felt they would be able to access care either at an emergency room or through other programs (though no specific programs were referenced). Qualitative responses suggest that participants generally felt safe at home as a sexual or gender minority person. Participants often explained that by staying home they were not putting themselves at risk for COVID-19, which in turn, made them feel safe. Further, many participants said that they were able to choose with whom they wanted to quarantine with and therefore felt comfortable to be themselves at home. While most participants only reported the negative health and economic impact of the quarantine (described in detail below), some participants reflected on positive things that emerged from the SAH order. Examples included an appreciation for the additional time with family and/or their partner, spending more time in nature, as well as reading, cooking, and doing other usually neglected projects around the house. Of note, no substantial differences were noted between LSMM and LTGW.

### Health and healthcare access

Quantitative responses to the EPII document a range of health and healthcare access-related concerns (Table 2). Results show nearly a quarter reported an increase in alcohol consumption (23.1%), however there was not a large increase in the reported use of other substances (3.9%). A substantial proportion of participants reported an increase in mental health problems for themselves (78.4%) or for a person in their home (17.7%). There were also large reported increases in sleep problems for themselves (67.3%) or for a person in their home (13%). Regarding healthcare access, 13.5% said they were unable to get needed medications and 34.6% said they got less medical attention than usual. When asked specifically about PrEP (Table 3), more than one-third (35.6%) reported being on PrEP prior to COVID-19; among those previously taking PrEP, 66.7% have continued taking it and reported getting refills either via telemedicine appointments or office-based visits. One-quarter (25.0%) reported being either concerned or very concerned about their ability to access PrEP during the SAH order.

The qualitative responses echoed the overall impact of COVID-19 on the health and healthcare access of participants (Table 4). Most participants referenced the ways in which COVID-19 negatively impacted their mental health, especially given the ongoing economic uncertainty resulting from the pandemic. While some participants expressed how quarantine brought them closer with friends and family, others described how quarantine led to a sense of loneliness and isolation. We probed on healthcare access with respect to PrEP and HIV/STI testing. Regarding PrEP, the reasons for discontinuing PrEP related to a substantial decrease in the number of their sexual partners, so participants no longer felt like they needed to take it. A few participants drew parallels with HIV, saying they viewed COVID-19 as a highly infectious version of HIV and wanted to avoid it, and therefore continued taking PrEP. Finally, when asked about access to HIV/STI testing, respondents reported substantial confusion. Many participants did not know which HIV/STI testing centers were open during the SAH. There were mixed responses regarding the perceived risk of COVID-19 infection if they had to leave their home to get tested for HIV/STIs: some said that if they needed to get tested for HIV/STIs, they felt confident that clinics were putting appropriate measures in place to reduce their risk of contracting COVID-19. However, a majority of participants said they were concerned about the potential for becoming infected with COVID-19 while accessing HIV/STI testing.

This was the only domain were differences qualitatively emerged between LSMM and LTGW. Specifically, transgender participants raised unique concerns associated with their ability to access gender-affirming hormones and procedures. While several found no difficulty in continuing to access their hormones, others said that since the SAH, they experienced an increase in price for their hormones. LTGW also reported that elective surgeries, such as facial feminization procedures, or breast replacement for a previously botched surgery, were delayed.

### Employment and economic impact of COVID-19

The quantitative responses to the EPII confirm that the financial impact of COVID-19 was devastating (Table 2): Half reported losing their jobs (50.0%) or having work hours reduced or being furloughed (57.7%). More than one-third reported having to continue working despite being in contact with people who might be infected with COVID-19 (34.6%). Because of lost income, many were unable to get enough food (36.5%) or pay important bills like rent and utilities (55.8%). The participants’ substantial financial concerns are also reflected in their qualitative responses (Table 4). As referenced above, the financial uncertainty made some participants feel anxious, often keeping them up at night. One LSMM participant was aware that he was using alcohol as a crutch to help relax even though he knew it was an unhealthy way for him to cope with the financial stress. Here, documentation status appeared to play the most prominent role in the narratives told by participants. Specifically, many who lost their jobs said they didn’t qualify for unemployment or the stimulus check because they were undocumented.

## Discussion

Though no confirmed cases were noted, COVID-19 dramatically impacted other aspects of health and overall wellbeing of LSMM and LTGW. While research is generally limited, we explore similarities and differences with other published studies.

With respect to COVID-19 infection history and quarantine experience, a recent large online survey of sexual minority men across the U.S. (n = 1051) reported more symptoms of COVID (37.8%), and–similar to our study–reported a negligible number of confirmed COVID-19 cases (0.1%) [29]. Another small study also conducted among LSMM in Miami-Dade County (n = 12) suggests that despite low rates of confirmed COVID-19 cases among the sample, there is still fear and anxiety about spreading the infection [17]. Far more research is needed to more accurately understand the epidemiological landscape of COVID-19 infections, particularly in racial and ethnic communities. With respect to quarantining experiences, to our knowledge, there is no data reflecting the impact of SAH on conflict and arguments among LGBT communities. The World Health Organization made a statement in April 2020 highlighting a global increase in the reported rates of domestic violence since the COVID-19 outbreak began; however, it exclusively focused on violence against women [30]. Our data suggest participants were able to safely quarantine, though potential data collection challenges (e.g., transparent reporting of violence if quarantining with a perpetrator) may undermine our findings, and underscore the urgent need for additional research.

Our results suggest that the toll of COVID-19 on the health and healthcare access of LSMM and LTGW is substantial. Study findings highlight increases in alcohol use, as well as mental health challenges and sleep problems. These results are largely consistent with the limited data that are currently available [29, 17]. The aforementioned national study did, however, also note an increase in drug use, a finding that did not bear out in our own study. Our results also highlighted disruptions in PrEP persistence. Other studies have noted similar disruptions in general health, and specifically for HIV prevention and treatment services, and suggest they may be further exacerbated as the pandemic continues [31–33]. While our results underscore the need for improved messaging around the availability of HIV testing, innovative ways to provide critical HIV prevention services are evolving: Home specimen collection kits and self-tests are being made available and telehealth visits (supported by HIPPA compliant technology), accompanied with longer prescription supplies, support continued PrEP use that are but a few examples [6]. However, efforts to appropriately scale these efforts require addressing several potential barriers such as achieving payment parity for telemedicine assessments, develop a unique workflow that clearly identifies if and when an in-person visit is required as well as what labs can (or cannot) be delayed [34]. While the results reported here highlight concerns that require focused responses to address PrEP disruptions, they also suggest potential ways for services to adapt going forward (e.g., telehealth). Of note, the main substantial difference between LSMM and LTGW emerged with respect to accessing healthcare for gender affirming hormones and procedures, an issue highlighted elsewhere [35, 36]. While efforts to determine what should be defined as ‘essential procedures’ are impacting a range of often politicized domains of care (e.g., access to pregnancy termination services), additional studies are needed to confirm the extent of this problem.

The financial impact of COVID-19 was widespread and is likely to have long-lasting affect: Half of those we interviewed reported job loss, and more than one third reported substantial challenges purchasing food and paying bills. Participants often referenced how the economic concerns negatively impacted their mental health and ability to sleep. The economic vulnerability of undocumented Latinx communities, and especially among those who identify as sexual and gender minorities, has already been noted [10]. Here, documentation status appeared particularly relevant for experiencing financial hardships and calls into question how an entire class of workers who contribute significantly to the economy can be supported at this unprecedented time [37]. Certain companies have taken steps to reduce payments (e.g., internet, electricity) [38] or provide free access to internet for a limited time [39]. Many of these efforts are impromptu, with an evolving list of resources being made publicly available [40]. Questions remain how these and other efforts can be tailored to address the unique needs faced by racial and ethnic minority LGBT communities such as LSMM and LTGW.

To our knowledge, this study is one of the first to provide empirical data regarding the wide range of health and wellbeing challenges faced by LSMM and LTGW during the SAH in response to COVID-19. However, the study has limitations. Our sample was based on participants from the parent study, however as noted elsewhere [41], change in phone numbers and non-response was common and may introduce selection bias (e.g., those facing a loss of employment may have more time to answer the phone, though conversely may lead someone to feeling sad or depressed and unwilling to answer their phone). While our sample size provides substantial depth for the qualitative analysis, we were limited in the range of statistical tests we could conduct with the quantitative data because of sample size. Further, though the mixed-methods design generally allowed for triangulation, it is possible that the qualitative responses with respect to their reported safety at home were limited due to an unwillingness of participants to expand on potential conflict or violence at home beyond their initial ‘yes’ or ‘no’ response to the quantitative question.

## Conclusions

Though no infections were noted, COVID-19 has impacted other aspects of health and overall wellbeing of LSMM and LTGW. Public health responses to COVID-19 should address stressors noted in other general population studies (e.g., mental health concerns) as well as other stressors that may disproportionately impact LGBT communities (e.g., continued PrEP use, access to HIV/STI testing, and body affirming hormones and procedures for transgender women in particular). Further, a comprehensive response is needed to address individual-, programmatic- and structural-level barriers faced by LSMM and LTGW. For example, campaigns addressing individual behaviors to reduce the potential for contracting COVID-19 (e.g., extensive handwashing, physical distancing, and mask wearing) should not only be available in Spanish and represent a range of skin tones, but also include diverse relationships beyond heteronormative family structures. Programmatic-level action steps highlight how trusted community partners can play a vital role by helping to navigate the quickly changing policy landscape with regard to state [42] and federal aid [43] during this pandemic. Further, they can clarify ambiguities regarding SAH orders to explain which essential services (such as HIV testing) continue to be available, noting the time and location of such clinics. Structural-level action steps are simultaneously needed. For example, extensive steps should be taken to ensure aggressive immigration tactics–e.g., ICE targeting hospitals or clinics–are halted during the pandemic and beyond [14]. These efforts to address issues raised by COVID-19 can move forward in conjunction with ongoing endeavors to bolster non-discrimination protections and access to federally funded programs that have largely been undermined by current legislation. Across these levels, researchers have the responsibility to use measures that accurately assess race/ethnicity as well as sexual orientation and gender identity to ensure we appropriately document the extent to which COVID-19 impacts these communities [44], and with concerted action, can also track our progress at mitigating the pandemic’s devastating impact across Latinx LGBT communities. This study contributes to the growing base of evidence regarding the ways in which COVID-19 will exacerbate existing disparities and also provides concrete suggestions to move us beyond documenting these disparities and instead, actively address them.
